# Absolute Reticulocyte Count and Reticulocyte Hemoglobin Content as Predictors of Early Response to Exclusive Oral Iron in Children with Iron Deficiency Anemia

**DOI:** 10.1155/2016/7345835

**Published:** 2016-03-22

**Authors:** Emilia Parodi, Maria Teresa Giraudo, Fulvio Ricceri, Maria Luigia Aurucci, Raffaela Mazzone, Ugo Ramenghi

**Affiliations:** ^1^Pediatric and Neonatology Unit, Ordine Mauriziano Hospital, Largo Turati 62, 10128 Turin, Italy; ^2^Department of Mathematics “G. Peano”, University of Turin, Via Carlo Alberto 10, 10123 Turin, Italy; ^3^Unit of Epidemiology, Regional Health Service ASL TO3, Via Sabaudia 164, Grugliasco, 10095 Turin, Italy; ^4^Hematology Unit, Department of Sciences of Public Health and Pediatrics, University of Turin, Piazza Polonia 94, 10126 Turin, Italy; ^5^Hematology and Coagulation Laboratory, Città della Scienza e della Salute Hospital, Piazza Polonia 94, 10126 Turin, Italy

## Abstract

We report data regarding kinetic of response to oral iron in 34 iron deficiency anemia children. Twenty-four/34 patients (70.5%) reached reference value of hemoglobin (Hb) concentration for age and sex at day + 30 from the beginning of treatment (complete early responders (CERs)), and 4/34 (12%) reached an Hb concentration at least 50% higher than the original (partial early responders (PERs)). CHr at T1 (within 7 days from the beginning of treatment) was significantly different in the different groups (22.95 in CERs versus 18.41 in other patients; *p* = 0.001; 22.42 in early responders versus 18.07 in NERs; *p* = 0.001). Relative increase of CHr from T0 to T1 resulted significantly higher in CERs than in other patients (0.21 versus 0.11, *p* = 0.042) and in early responders than in NERs (0.22 versus 0.004, *p* = 0.006). Multivariate logistic models revealed a higher probability of being a complete early responder due to relative increase of ARC from T0 to T1 [OR (95% CI) = 44.95 (1.54–1311.98)] and to CHr at T1 [OR (95% CI) =3.18 (1.24–8.17)]. Our preliminary data confirm CHr as early and accurate predictor of hematological response to oral iron.

## 1. Introduction

Reticulocytes are the youngest erythrocytes released from the bone marrow into circulating blood. Under normal conditions, after maturing for 1–3 days within the bone marrow, they are released into peripheral blood where they circulate for 1-2 days before becoming mature erythrocytes. The reticulocyte hemoglobin content (CHr) provides an indirect measure of the functional iron available for new red blood cell production over the previous 3-4 days [1]. CHr in peripheral blood samples has been proven to be a useful marker for diagnosis of iron deficiency and iron deficiency anemia (IDA) both in children [2–4] and adults [5].

Brugnara et al. [6] demonstrated that CHr also provides an early measure of the response to parenteral iron therapy increasing within 2–4 days of the initiation of intravenous iron therapy.

The goal of therapy for IDA, the most common hematological disease of infancy and childhood, is to supply sufficient iron to repair the hemoglobin (Hb) deficit and replenish storage iron [7]. Oral iron administration is a well-established effective and worldwide accepted treatment for anemia derived from inadequate dietary iron intake because of its efficacy, safety, and cost-effectiveness [8]. Thus, in our Pediatric Hematology Unit, oral iron treatment is the first-line therapy for all children with IDA with anamnestic suspicion of inadequate iron intake, independently of base Hb level. Recently, we have identified absolute reticulocyte count (ARC) and CHr as accurate and precocious markers in order to early detect early responders to exclusive oral iron therapy in a small cohort of pediatric patients with severe iron deficiency anemia (IDA) [9]. These preliminary results in children with very low base Hb levels (median Hb level before treatment 6.3 g/dL; range 4.5–7.0) prompted us to investigate the efficacy of reticulocyte parameters in monitoring the response to oral iron supplementation in a larger cohort of pediatric patients.

## 2. Materials and Methods

### 2.1. Patients and Hematological Evaluation

Clinical records of patients referred to our Pediatric Hematology Unit for IDA between July 1, 2012, and June 30, 2014, were retrospectively analyzed.

Patients who matched inclusion criteria and did not present exclusion criteria were included in the study. Data about patients with an age between 6 months and 16 years, a hemoglobin level more than 2 standard deviations below the mean reference value for age and gender, a transferrin saturation < 15%, and a high anamnestic suspicion of inadequate iron intake were analyzed. Patients with diagnosis of celiac disease, positivity of fecal occult blood test, or positivity of* Helicobacter pylori* fecal antigen test were excluded. Patients from our preliminary report were not included in the present study [9].

All children with these characteristics had started a first-line treatment with exclusive oral iron supplementation at a dosage equivalent to 2 mg/kg/die of elemental iron. In all patients but one, who underwent therapy with liposomial iron, bisglycinate chelate iron was administered.

Data about Hb levels and reticulocyte parameters (ARC and CHr) at diagnosis, before treatment (T0), within 7 days (T1), and at day +30 (T2) from the beginning of iron supplementation, were extrapolated from clinical records of each patient and analyzed with analytical methods.

Hb and reticulocyte parameters on peripheral blood samples had been measured with an automated flow cytometer (Advia 120 Bayer®) with optic measure; reticulocytes had been stained with dye oxazine 750. Approximately 50.000 cells had been counted for each red blood cell and reticulocyte determinations.

### 2.2. Statistical Analysis

We described data as number and frequencies or mean and standard deviation for qualitative and quantitative variables, respectively.

Differences between different groups of patients were tested using Fisher's exact test or Wilcoxon sum rank test (due to the not normal distribution of all the values as established by means of Shapiro-Wilks normality tests), as appropriate.

To account for possible confounding variables, we performed a multivariate logistic model, adjusting for age, sex, and Hb value at diagnosis.

Relative increase of ARC and CHr from T0 to T1 was computed as (T1  value − T0  value)/T0  value.

All tests were two-sided and a *p* value lower than 0.050 was considered significant. Analyses were performed using SAS V9.4.

### 2.3. Definitions

Response was defined as early and complete if patients reached mean Hb reference value for age at day +30 (T2) from the onset of iron therapy (complete early responders (CERs)).

Response at T2 was defined as early and partial if patients reached an Hb concentration at least 50% higher than the original one, computed as (Hb  T2 − Hb  T0)/Hb  T0 (partial early responders, PERs).

Patients who at T2 did not achieve an Hb concentration at least 50% higher than the original one were classified as not early responders (NERs).

## 3. Results and Discussion

### 3.1. Patients

Thirty-four pediatric patients, 22 males and 12 females, with a mean (SD) age at diagnosis of 52 (61) months, matched inclusion criteria and were enrolled in the study. Patients' characteristics at diagnosis are set out in Table 1.

### 3.2. Hemoglobin and Reticulocyte Parameters at T0, T1, and T2

Data regarding values of Hb, ARC, and CHr at T0, T1, and T2 are set out in Table 2.

Mean (SD) Hb level before treatment (T0) was 6.84 (1.22) g/dL. It increased to 7.36 g/L (1.31) and 10.56 g/L (1.62) at T1 and T2, respectively.

Mean (SD) ARC before treatment (T0) was 72799/mmc (35155). It increased to 168583/mmc (101196) at T1 and then decreased again to 76317/mmc (38924) at T2, respectively.

CHr could be fully monitored only for 28 patients. Mean CHr before treatment was 18.20 pg (2.32). It increased to 21.48 pg (3.04) and 25.43 pg (4.49) at T1 and T2, respectively.

Twenty-four/34 patients (70.5%) were classified as complete early responders (CERs), 4/34 (12%) as partial early responders (PERs), and the remaining 6 (17.5%) as not early responders (NERs).

Data about mean Hb, ARC, and CHr at T0, T1, and T2 in different groups of patients are set out in Tables 3(a) and 3(b).

**Figure 1:**
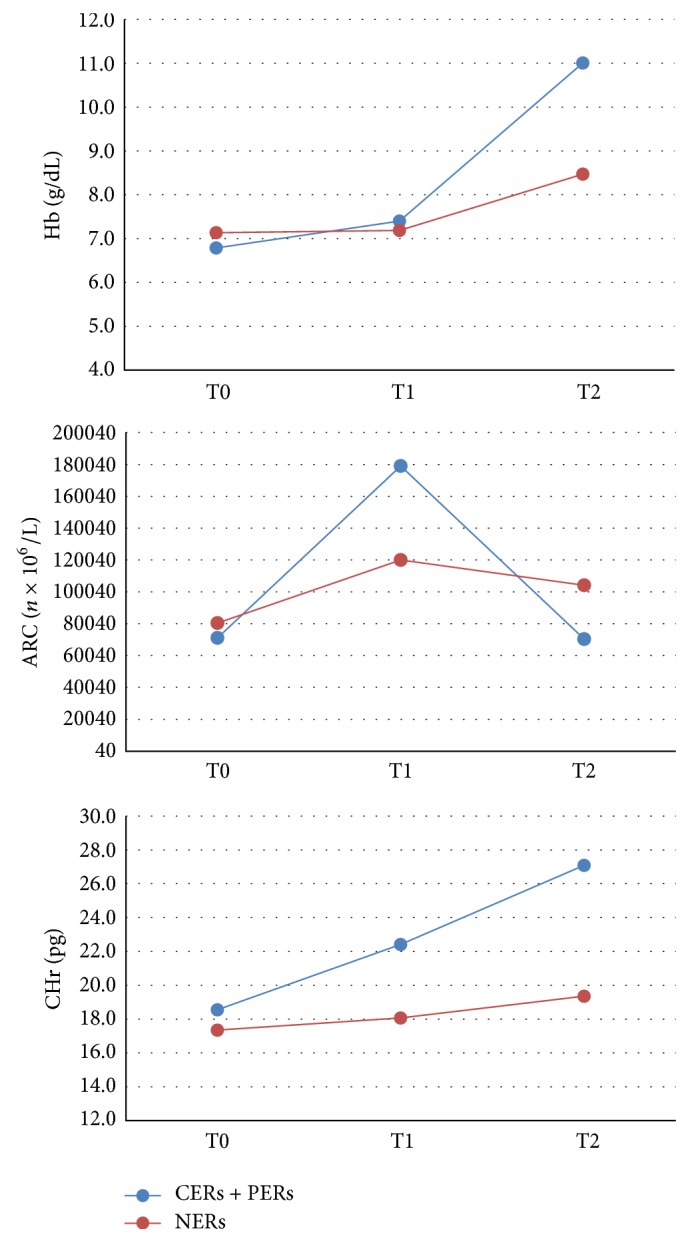
Trends of mean Hb, ARC, and CHr values (*y*-axis) in early responding patients (CERs + PERs) and not early responding patients (NERs) at T0: diagnosis, T1: within 7 days from the beginning of oral iron supplementation, and T2: at +30 from the onset of therapy (*x*-axis).

Trends of mean values of Hb, ARC, and CHr in the different groups of children are graphically represented in Figure 1.

### 3.3. Factors Predictive of Response at Day +30: Comparison between Complete Early Responders and Other Patients

No differences were detected regarding gender and hemoglobin values at diagnosis (T0).

CHr at T0 was significantly higher in CERs than in remaining patients (19.94 pg versus 16.73 pg; *p* = 0.020).

Analysis of Hb values at T1 revealed no difference between the two different groups (7.67 g/dL versus 6.63 g/dL, *p* = 0.05).

CHr at T1 was significantly different between CERs and other patients (22.95 versus 18.41; *p* = 0.001).

Relative increase of CHr from T0 to T1, computed as (T1  CHr − T0  CHr)/T0  CHr, resulted significantly higher in CERs than in other patients (0.21 versus 0.11, *p* = 0.042).

Results from multivariate logistic models revealed a higher probability of being a complete early responder due to relative increase of ARC from T0 to T1 [OR (95% CI) = 44.95 (1.54–1311.98); *p* < 0.030] and to CHr at T1 [OR (95% CI) = 3.18 (1.24–8.17); *p* = 0.016]. Both models were adjusted by age at diagnosis, sex, and level of Hb at T0.

Differences between CERs and other patients are reported in Table 3(a).

### 3.4. Factors Predictive of Response at Day +30: Comparison between Early Responders and Not Early Responders

When the distinction between early responders and not responders was taken into account, CHr at T1 was significantly different in the two groups (22.42 versus 18.07; *p* = 0.001).

Relative increase of CHr from T0 to T1, computed as (T1  CHr − T0  CHr)/T0  CHr, resulted significantly higher, too (0.22 versus 0.004, *p* = 0.006).

Differences between early responders and not responders are reported in Table 3(b).

### 3.5. Discussion

Our data confirm the efficacy of oral iron administration in promptly improving Hb values in patients with severe anemia, too.

More than 70% of patients retrospectively enrolled in the study reached mean hemoglobin (Hb) reference value for age and gender within one month from the initiation of treatment, independently from base Hb level. Only a negligible percentage of children did not reach an Hb concentration at least 50% higher than the original after one month of therapy and was therefore classified as unresponsive to therapy. As our patients have not been tested for alpha and beta thalassemia at diagnosis or during the first month of therapy, we cannot rule out the possibility that these children have not responded to iron because of a concomitant hemoglobinopathy.

Measurement of reticulocyte hemoglobin content (CHr) has been validated in literature as the strongest independent predictor of iron deficiency and iron deficiency anemia, when compared to other laboratory markers (hemoglobin, ferritin, transferrin saturation, or mean corpuscular volume), both in children and in adults [1]. In pediatric patients, optimal CHr cut-off has been proven to be 27.5 pg for detecting iron deficiency (with a sensitivity of 83% and a specificity of 72%) [3] and 26 pg for detecting iron deficiency anemia (with a sensitivity of 83% and a specificity of 75%) [2]. In the totality of children described in our cohort (all displaying severe iron deficiency, defined by a transferrin saturation value of less than 10%), CHr levels were significantly lower than cut-off level of 26 pg (*p* < 0.001 for corresponding Wilcoxon test), and in 75% of children resulted even less than 20 pg.

The finding that CHr at T0 was significantly lower in patients who did not reach normal Hb levels at day +30 raises the intriguing possibility that CHr level at diagnosis might be a predictor of early response to oral iron. To our knowledge, no data about this issue are present in literature to date. Due to the limitations derived from the small sample size, our results need to be confirmed on larger cohorts of patients.

As reticulocyte indices allow a real-time evaluation of iron deficient erythropoiesis and of the effectiveness of iron replacement therapy [1], CHr has been validated in literature as an early predictor of response to parenteral iron therapy, in children receiving hemodialysis [10] and in adults [6].

Similar conclusion regarding response to exclusive oral iron therapy in a small cohort of children with severe iron deficiency anemia had been previously reported by our group [9]. Main outcome of our preliminary study [9] was to evaluate if oral iron supplementation should be proposed as first-line treatment in a small cohort of clinically asymptomatic patients with severe iron deficiency anemia. These patients, due to their Hb levels (median 6.3 g/dL, range 4.5–7), needed a tight follow-up; for that reason, the kinetic of response was assessed within 48 hours from the onset of iron administration (T1) and our data demonstrated the utility of ARC and CHr as accurate markers for the early detection of patients not responding to oral iron to be quickly switched to other therapies (parenteral iron supplementation or transfusion). These results prompted us to investigate the efficacy of reticulocyte parameters in monitoring the response to oral iron supplementation in a larger cohort of pediatric patients.

Despite the limitations derived from the retrospective nature of the study, analysis of reticulocyte parameters within one week from the beginning of therapy confirmed CHr to predict good early hematologic response to iron supplements after one week of therapy.

In particular, both CHr levels within 7 days from the beginning of therapy and the relative increase of CHr from T0 to T1 resulted significantly higher in patients who displayed normal hemoglobin levels or a hemoglobin concentration at least 50% higher than the original one at day +30.

## 4. Conclusions

Aim of the present study was to evaluate kinetic of response to oral iron in a cohort of pediatric patients with iron deficiency anemia and to investigate the usefulness of reticulocyte parameters CHr and ARC in monitoring early response to iron supplementation.

Despite the limitations derived from both the small number of patients and the retrospective nature of the study, according to data of literature, our data confirm that oral iron supplementation is an effective treatment for iron deficiency anemia, independently from base hemoglobin at diagnosis.

Moreover, our preliminary data suggest that reticulocyte parameters are early and accurate predictors of response to oral therapy and that pediatricians could take advantage of reticulocyte hemoglobin content both at diagnosis and during follow-up of iron deficiency anemia for assessing early erythropoietic response to iron replacement therapy.

A prospective multicentre study on a larger cohort of patients is ongoing in order to reinforce our conclusions.

## Figures and Tables

**Table 1:** Patients' characteristics at diagnosis.

Patient (id)	Sex (M/F)	Age at diagnosis (months)	Hb T0 (g/dL)	ARC T0 (*n*/mmc)	CHr T0 (pg)
1	M	9	5.8	82800	15.3
2	M	9	5.5	79800	15.4
3	M	12	5.1	54100	15.4
4	M	48	5.0	82200	15.7
5	M	7	5.7	148100	15.7
6	M	24	4.5	145700	15.8
7	F	24	6.8	89200	16.1
8	F	24	6.1	66700	16.4
9	F	144	7.0	126000	16.7
10	F	96	6.3	92300	16.9
11	F	72	6.3	85400	17.2
12	M	36	7.9	78300	17.5
13	M	48	8.1	94000	18.0
14	M	7	8.7	43700	18.2
15	M	9	6.0	56800	18.2
16	F	36	7.8	69500	18.5
17	F	7	5.8	78000	18.8
18	M	11	9.3	53600	18.8
19	F	8	8.9	15500	19.0
20	F	180	7.4	63500	19.4
21	M	60	7.2	47700	19.7
22	M	192	8.5	40500	20.4
23	M	24	7.4	46600	20.4
24	F	156	6.8	78600	20.5
25	F	180	6.5	56100	20.6
26	M	48	7.9	59700	21.2
27	M	36	8.0	19800	21.8
28	M	4	8.8	57700	24.8
29	M	192	6.3	68000	na
30	M	24	6.7	18600	na
31	M	6	6.3	132000	na
32	M	24	6.0	152000	na
33	M	5	6.8	50000	na
34	F	8	5.5	42000	na

**Table 2:** Hemoglobin and reticulocyte parameters at T0 (before treatment), T1 (within 7 days from the beginning of oral iron), and at T2 (day +30 from the beginning of treatment).

Patient (id)	T0 Hb (g/dL)	T1 Hb (g/dL)	T2 Hb (g/dL)	T0 ARC (*n*/mmc)	T1 ARC (*n*/mmc)	T2 ARC (*n*/mmc)	T0 CHr (pg)	T1 CHr (pg)	T2 CHr (pg)
1	5.8	6.3	9.1	82800	182200	84200	15.3	21.0	26.0
2	5.5	5.7	8.4	79800	137300	141300	15.4	15.8	19.6
3	5.1	5.9	11.1	54100	99100	44000	15.4	18.7	29.8
4	5.0	4.9	7.0	82200	126300	78900	15.7	16.8	16.4
5	5.7	5.9	8.0	148100	232000	154100	15.7	17.5	17.7
6	4.5	4.5	9.4	145700	209400	122200	15.8	20.5	24.9
7	6.8	7.6	12.9	89200	190000	47400	16.1	21.9	25.2
8	6.1	7.2	13.2	66700	155300	31500	16.4	24.2	30.1
9	7.0	7.2	12.4	126000	201400	106200	16.7	19.9	29.0
10	6.3	7.1	11.8	92300	315000	79400	16.9	22.3	27.2
11	6.3	6.1	7.4	85400	134500	107000	17.2	18.3	19.3
12	7.9	8.1	9.8	78300	110600	66000	17.5	20.8	23.7
13	8.1	8.6	10.5	94000	148200	54300	18.0	23.8	27.6
14	8.7	9.7	9.4	43700	71600	67100	18.2	18.8	18.1
15	6.0	7.0	12.2	56800	108200	60000	18.2	19.7	26.6
16	7.8	7.4	9.5	69500	71500	69500	18.5	19.3	23.6
17	5.8	6.3	12.4	78000	232000	43800	18.8	21.0	31.3
18	9.3	9.1	9.5	53600	84200	148300	18.8	17.7	21.0
19	8.9	9.3	10.5	15500	262500	61900	19.0	25.6	22.5
20	7.4	8.8	10.8	63500	148100	44800	19.4	27.3	27.6
21	7.2	7.2	12.5	47700	155900	71100	19.7	22.7	28.9
22	8.5	8.4	10.5	40500	44600	70100	20.4	25.7	26.2
23	7.4	8.8	10.5	46600	240300	42200	20.4	26.7	32.1
24	6.8	7.7	13.7	78600	105500	26100	20.5	24.3	32.0
25	6.5	8.9	10.7	56100	149900	188500	20.6	22.6	22.3
26	7.9	7.5	10.4	59700	93900	35400	21.2	22.0	26.3
27	8.0	8.9	11.8	19800	42000	63500	21.8	22.9	30.9
28	8.8	9.2	10.8	57700	59200	75500	24.8	23.9	26.2
29	6.3	6.9	10.0	68000	101340	82800	na	na	na
30	6.7	8.0	10.0	18600	345700	26400	na	na	na
31	6.3	6.7	12.4	132000	294100	37000	na	na	na
32	6.0	6.7	9.3	152000	233000	103000	na	na	na
33	6.8	6.7	9.8	50000	110000	94500	na	na	na
34	5.5	6.0	11.2	42000	537000	66800	na	na	na

**(a):** 

	CERs (*n* = 24)		PERs + NERs (*n* = 10)		
	*n*	(%)	*n*	(%)	*p* value

Male	14	(63.64%)	8	(36.36%)	0.430
Female	10	(83.33%)	2	(16.67%)	

	Mean	(SD)	Mean	(SD)	*p* value

T0 Hb (g/dL)	7.00	(1.03)	6.46	(1.60)	0.134
T1 Hb (g/dL)	7.67	(1.02)	6.63	(1.68)	0.050
T2 Hb (g/dL)	11.33	(1.15)	8.70	(0.94)	<0.001

T0 ARC (*n*/mmc)	63820	(29399)	94280	(39813)	0.040
T1 ARC (*n*/mmc)	177077	(113504)	148200	(63023)	0.620
T2 ARC (*n*/mmc)	63300	(33954)	107560	(32749)	0.002
(T1 ARC − T0 ARC)/T0 ARC	*2.92*	*(4.87)*	*0.58*	*(0.29)*	*0.023 *

T0 CHr (pg)	19.04^*∗*^	(2.33)	16.73°	(1.44)	0.020
T1 CHr (pg)	22.95^*∗*^	(2.38)	18.41°	(1.69)	0.001
T2 CHr (pg)	27.66^*∗*^	(2.99)	20.73°	(3.38)	0.001
(T1 CHr−T0 CHr)/T0 CHr	*0.21* ^*∗*^	*(0.14)*	*0.11*°	*(0.14)*	*0.042 *
	^*∗*^ *n* = 19		°*n* = 9		

**(b):** 

	CERs + PERs (*n* = 28)		NERs (*n* = 6)		
	*n*	(%)	*n*	(%)	*p* value

Male	18	(81.82%)	4	(18.18%)	1.000
Female	10	(83.33%)	2	(16.67%)	

	Mean	(SD)	Mean	(SD)	*p* value

T0 Hb (g/dL)	6.78	(1.12)	7.13	(1.73)	0.790
T0 Hb (g/dL)	7.40	(1.20)	7.18	(1.90)	0.750
T0 Hb (g/dL)	11.00	(1.35)	8.47	(1.14)	0.001

T0 ARC (*n*/mmc)	71143	(35210)	80417	(36866)	0.580
T0 ARC (*n*/mmc)	178991	(105758)	120017	(61270)	0.120
T0 ARC (*n*/mmc)	70354	(36863)	104150	(39149)	0.040
(T1 ARC−T0 ARC)/T0 ARC	*2.60*	*(4.56)*	*0.49*	*(0.23)*	*0.005*

T0 CHr (pg)	18.56^*∗*^	(2.49)	17.35°	(1.39)	0.300
T1 CHr (pg)	22.42^*∗*^	(2.73)	18.07°	(0.91)	0.001
T2 CHr (pg)	27.09^*∗*^	(3.30)	19.35°	(2.60)	<0.001
(T1 CHr−T0 CHr)/T0 CHr	*0.22* ^*∗*^	*(0.14)*	*0.04*°	*(0.06)*	*0.006 *
	^*∗*^ *n* = 22		°*n* = 6		

## References

[1] Piva E., Brugnara C., Spolaore F., Plebani M. (2015). Clinical utility of reticulocyte parameters. *Clinics in Laboratory Medicine*.

[2] Brugnara C., Zurakowski D., DiCanzio J., Boyd T., Platt O. (1999). Reticulocyte hemoglobin content to diagnose iron deficiency in children. *The Journal of the American Medical Association*.

[3] Ullrich C., Wu A., Armsby C. (2005). Screening healthy infants for iron deficiency using reticulocyte hemoglobin content. *The Journal of the American Medical Association*.

[4] Bakr A. F., Sarette G. (2006). Measurement of reticulocyte hemoglobin content to diagnose iron deficiency in Saudi children. *European Journal of Pediatrics*.

[5] Mast A. E., Blinder M. A., Dietzen D. J. (2008). Reticulocyte hemoglobin content. *American Journal of Hematology*.

[6] Brugnara C., Laufer M. R., Friedman A. J., Bridges K., Platt O. (1994). Reticulocyte hemoglobin content (CHr): early indicator of iron deficiency and response to therapy. *Blood*.

[7] Borgna-Pignatti C., Marsella M. (2008). Iron deficiency in infancy and childhood. *Pediatric Annals*.

[8] Zeng X., Wu T. (2007). Iron supplementation for iron deficiency anemia in children. *Cochrane Database of Systematic Reviews*.

[9] Parodi E., Giraudo M. T., Davitto M. (2012). Reticulocyte parameters: markers of early response to oral treatment in children with severe iron-deficiency anemia. *Journal of Pediatric Hematology/Oncology*.

[10] Warady B. A., Kausz A., Lerner G. (2004). Iron therapy in the pediatric hemodialysis population. *Pediatric Nephrology*.

